# A disproportionality analysis of FDA adverse event reporting system (FAERS) events for ticagrelor

**DOI:** 10.3389/fphar.2024.1251961

**Published:** 2024-04-09

**Authors:** Yunyan Pan, Yu Wang, Yifan Zheng, Jie Chen, Jia Li

**Affiliations:** ^1^ Department of Pharmacy, The First Affiliated Hospital of Sun Yat-sen University, Guangzhou, China; ^2^ School of Pharmaceutical Sciences, Sun Yat-sen University, Guangzhou, China; ^3^ Department of Clinical Pharmacy Translational Science, University of Michigan College of Pharmacy, Ann Arbor, MI, United States

**Keywords:** ticagrelor, adverse events, FDA adverse event reporting system, disproportionality analysis, data mining

## Abstract

**Background:**

Ticagrelor is a commonly used antiplatelet agent, but due to the stringent criteria for trial population inclusion and the limited sample size, its safety profile has not been fully elucidated.

**Method:**

We utilized OpenVigil 2.1 to query the FDA Adverse Event Reporting System database and retrieved reports by the generic name “ticagrelor” published between 1 October 2010 and 31 March 2023. Adverse drug events (ADEs) were classified and described according to the preferred terms and system organ classes in the Medical Dictionary of Regulatory Activity. Proportional reporting ratio (PRR), reporting odds ratio (ROR) and Bayesian Confidence Propagation Neural Network (BCPNN) were used to detect signals.

**Results:**

The number of ADE reports with ticagrelor as the primary suspect drug was 12,909. The top three ADEs were dyspnea [1824 reports, ROR 7.34, PRR 6.45, information component (IC) 2.68], chest pain (458 reports, ROR 5.43, PRR 5.27, IC 2.39), and vascular stent thrombosis (406 reports, ROR 409.53, PRR 396.68, IC 8.02). The highest ROR, 630.24, was found for “vascular stent occlusion”. Cardiac arrest (137 reports, ROR 3.41, PRR 3.39, IC 1.75), atrial fibrillation (99 reports, ROR 2.05, PRR 2.04, IC 1.03), asphyxia (101 reports, ROR 23.60, PRR 23.43, IC 4.51), and rhabdomyolysis (57 reports, ROR 2.75, PRR 2.75, IC 1.45) were suspected new adverse events of ticagrelor.

**Conclusion:**

The FAERS database produced potential signals associated with ticagrelor that have not been recorded in the package inserts, such as cardiac arrest, atrial fibrillation, asphyxia, and rhabdomyolysis. Further clinical surveillance is needed to quantify and validate potential hazards associated with ticagrelor-related adverse events.

## 1 Introduction

P2Y12 inhibitors, such as clopidogrel, prasugrel, and ticagrelor, have emerged as pivotal in mitigating adverse cardiovascular outcomes following revascularization in coronary artery disease (CAD). Among them, ticagrelor is a third-generation P2Y12 receptor antagonist that reversibly binds to the P2Y12 receptor and almost completely inhibits ADP-induced platelet aggregation *in vitro* (Husted et al., 2006). Ticagrelor was approved for marketing by the European Medicines Agency (EMA) on 3 December 2010, followed by the US Food and Drug Administration (FDA) on 20 July 2011. A genetic sub-study in the Platelet inhibition and patient Outcomes (PLATO) trial showed that CYP2C19 or ABCB1 gene diversity did not affect the efficacy of ticagrelor in reducing major cardiovascular events compared to clopidogrel (Wallentin et al., 2010). Several guidelines recommend ticagrelor as the first-line or preferred antiplatelet agent for patients with acute coronary syndrome (ACS) (Amsterdam et al., 2014; Collet et al., 2021). A study based on the United States databases showed that clopidogrel is the most used P2Y12 inhibitor, accounting for 60.9% of the prescription share, followed by ticagrelor (25.1%) and prasugrel (13.6%). For patients less than or equal to 65 years, ticagrelor use increased from 13.7% in 2013 to 45.6% in 2018 and exceeded clopidogrel use in the third quarter of 2018 (Kumar et al., 2023). As the usage rate of ticagrelor increases their long-term safety profiles remain incompletely evaluated, posing potential risks to an increasing number of users.

Although the safety evaluation of ticagrelor has been conducted in clinical trials, due to the stringent criteria for trial population inclusion and the limited sample size, serious adverse drug events (ADEs) with low incidence and long-term medication safety issues cannot be clarified during the clinical trial phase. With the expansion of the user base of ticagrelor after its launch, the FDA has requested revisions and updates to the drug safety information of ticagrelor. In October 2019, the FDA approved a revision of the package insert for ticagrelor, which added new safety information about thrombotic thrombocytopenic purpura (TTP) (FDA, 2019). TTP is a serious condition which can occur after a brief drug exposure (<2 weeks) and requires prompt treatment (George, 2022). Furthermore, new safety information about central sleep apnea (CSA) and Cheyne-Stokes respiration was added in September 2020, and a new section about CSA and Cheyne-Stokes respiration was added to the “WARNINGS AND PRECAUTIONS” section in August 2021 (FDA, 2020; FDA, 2021). Over the past years, several case reports have emerged showing the possibility of serious ticagrelor-induced bradyarrhythmia (Al-Bayati et al., 2021; Aranganathan et al., 2021; Kotaru and Kalavakunta, 2021). A sub-study of the PLATO trial showed that more patients treated with ticagrelor had ventricular pauses compared to clopidogrel-treated patients, but there were no apparent clinical consequences related to the increase in ventricular pauses in patients receiving ticagrelor (Scirica et al., 2011). To date, the risk of bradyarrhythmia in patients treated with ticagrelor is still incompletely evaluated. A meta-analysis showed that ticagrelor increased the risk of bradyarrhythmia or severe bradyarrhythmia; however, due to missing outcome data in two-thirds of eligible studies, the evidence was low to moderate (Pujade et al., 2020). A pharmacovigilance study compared the adverse drug reaction signals of ticagrelor and clopidogrel, but did not summarize ADEs related to arrhythmia (Tang et al., 2022). With the increase in the number of users of ticagrelor, it is still necessary to conduct post-market reassessment to characterize new and serious ADEs.

Our study aimed to conduct a pharmacovigilance analysis for ticagrelor and ADEs using the FDA Adverse Event Reporting System (FAERS) database to explore the post-marketing safety profile of ticagrelor.

## 2 Materials and methods

### 2.1 Data sources and collection

The FAERS database is a publicly available database that collects ADE reports spontaneously reported by healthcare professionals, patients, pharmaceutical manufacturers, etc., in different regions, and reflects the real-world occurrence of ADEs (Wei et al., 2023). Data mining algorithms have been used for safety monitoring and re-evaluation of drugs post-marketing from FAERS databases (Shu et al., 2022; Tian et al., 2022; Jiang et al., 2024). Therefore, we evaluated the safety of ticagrelor by analyzing the proportional imbalance of ADEs in the FAERS database since its launch.

Data for this study were obtained from the FAERS database. The AEs in the FAERS database were coded using Medical Dictionary for Regulatory Activities (MedDRA) terminology (https://www.meddra.org/). OpenVigil is a novel web-based pharmacovigilance analysis tool which uses the openFDA online interface of the FDA to access US pharmacovigilance data from the FAERS database (Böhm et al., 2012). OpenVigil 2.1 is an AE data extraction and cleaning, mining, and analysis tool specifically designed for the FAERS database, which currently includes the FAERS data from 1 January 2004 to 31 March 2023. OpenVigil relies on the U.S. Adopted Name (USAN) scheme, only valid reports with an unambiguous mapping of the free-text drug name to a USAN drug name were included in the analysis. In the FAERS database, there are numerous updates on cases, which means an entire case may include many unique reports. In this study, entire cases were used for analysis, a case contributes to the result if at least one of its reports includes the ticagrelor-event relationship.

We used OpenVigil 2.1 to query the FAERS database and retrieve reports on the generic name “ticagrelor” from 1 October 2010 to 31 March 2023. In each AE report from the FAERS database, the reporters assigned role codes for each reported drug. In our study, we selected cases defined as AE reports, in which the reporter referred to ticagrelor as a “Primary Suspect.” AEs were classified and described according to the preferred terms (PTs) and the system organ classes (SOCs) in the international MedDRA, version 24.0. Because it is impossible to identify individual patients, ethical approval was not required in our hospital.

### 2.2 Statistical analysis

Proportional reporting ratio (PRR), reporting odds ratio (ROR) and Bayesian Confidence Propagation Neural Network (BCPNN) methods are commonly used to detect ADE signals in pharmacovigilance (Noguchi et al., 2021). PRR can be used to estimate the relative risk, but the PRR method is sensitive and prone to false positive signals, especially when the number of reported cases is low, while ROR is a consistent estimate of the rate ratio or hazard ratio and is less biased than other indices. The advantage of BCPNN is that it is relatively stable even when the number of reports is small. Therefore, we combined ROR, PRR and BCPNN method to mine the ADE signals of ticagrelor, and when the results of the three methods were positive, the signal was judged to be a suspected ADE signal. The criteria of disproportionate measure and standard of signal detection were shown in (Table 1) (Bate et al., 1998; Evans et al., 2001; van Puijenbroek et al., 2003). In order to better demonstrate the strength of ADE signals, we defined the judgment criteria for signal intensity shown in Supplementary Table S1. The higher the PRR/ROR/IC value, the higher the strength of the signal.

**TABLE 1:** Formulas and signal detection criterias for reporting odds ratio (ROR), proportional reporting ratio (PRR) and bayesian confidence propagation neural network (BCPNN) (Shu et al., 2022; Jiang et al., 2024).

Algorithms	Equation	Criteria
ROR	$\text{ROR}=\left(a/c\right)/\left(b/d\right)$	a>3; the lower limit of 95%Cl > 1
	$\text{SE}\left(\;\ln\text{ ROR}\right)=\sqrt{\frac{1}{a}+\frac{1}{b}+\frac{1}{c}+\frac{1}{d}}$	
	$95\%CI=e^{\ln\left(ROR\right)\pm 1.96\sqrt{\frac{1}{a}+\frac{1}{b}+\frac{1}{c}+\frac{1}{d}}}$	
PRR	$PRR=\left[a/\left(a+b\right)\right]/\left[c/\left(c+d\right)\right]$	a>3; PRR>2χ²>4
	$\chi ²=\frac{{\left(ad-bc\right)}^{2}\times \left(a+b+c+d\right)}{\left(a+b\right)\;\left(c+d\right)\;\left(a+c\right)\;\left(b+d\right)}$	
BCPNN	$\text{IC}=\log_{2}\frac{p\left(x,y\right)}{p\left(x\right)p\left(y\right)}=\log_{2}\frac{a\left(a+b+c+d\right)}{\left(a+b\right)\left(a+c\right)}$	*a*>3; IC025 > 0
	$E\left(\text{IC}\right)=\log_{2}\frac{\left(a+\gamma 11\right)\left(a+b+c+d+\alpha \right)\left(a+b+c+d+\beta \right)}{\left(a+b+c+d+\gamma \right)\left(a+b+\alpha 1\right)\left(a+c+\beta 1\right)}$	
	$V\left(\text{IC}\right)=\frac{1}{{\left(\ln2\right)}^{2}}\left\{\left[[\frac{\left(a+b+c+d\right)\;-a+\gamma \;-\gamma 11}{\left(a+\gamma 11\right)\left(1+a+b+c+d+\gamma \right)}\right]]+\left[[\frac{\left(a+b+c+d\right)\;-\left(a+b\right)+\alpha -\;\alpha 1}{\left(a+b+\alpha 1\right)\left(1+a+b+c+d+\alpha \right)}\right]]+\left[[\frac{\left(a+b+c+d\right)\;-\left(a+c\right)+\beta \;-\beta 1}{\left(a+c+\beta 1\right)\left(1+a+b+c+d+\beta \right)}\right]]\right\}$	
	$\gamma =\gamma 11\frac{\left(a+b+c+d+\alpha \right)\left(a+b+c+d+\beta \right)}{\left(a+b+\alpha 1\right)\left(a+c+\beta 1\right)}$	
	$IC-2SD=E\left(IC\right)\;-2\sqrt{V\left(IC\right)}$	
	α1=β1=1,α=β=2,γ11=1	

Equation: *a,* number of reports containing both ticagrelor and the suspect adverse drug reaction; *b*, number of reports containing the suspect adverse drug reaction with other medications (except ticagrelor); *c*, number of reports containing ticagrelor with other adverse drug reactions (except the event of interest); *d*, number of reports containing other medications and other adverse drug reactions. ROR, reporting odds ratio; CI, confidence interval; PRR, proportional reporting ratio; χ^2^, chi-squared; BCPNN, bayesian confidence propagation neural network; IC, information component; IC025, the lower limit of 95%CI, of the IC.

## 3 Results

### 3.1 ADE reports and clinical information

A total of 9,699,440 ADE reports were identified from FAERS database from 1 October 2010 to 31 March 2023. There were 12,909 ADE reports with ticagrelor as the primary suspect drug, involving 2,229 PTs. The number of reports was much higher in males (7,421 reports, 57.49%) than in females (4,310 reports, 33.39%); the main age group was 65–84 years (3,302 reports, 25.58%); most reports were submitted in 2016 (3,203 reports, 24.81%); and the main reporting country was the US (8,058 reports, 62.42%) (Supplementary Table S2).

### 3.2 Signal ADE mining

**FIGURE 1:**
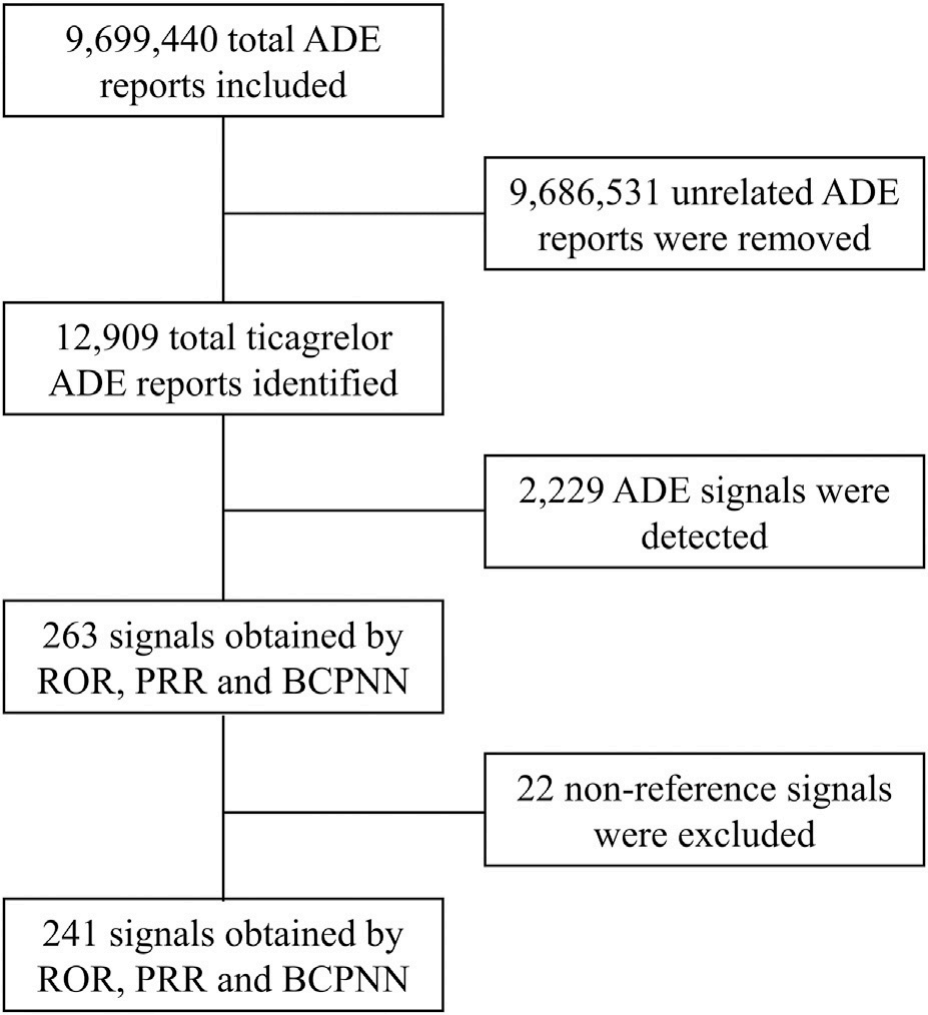
Flow chart for ticagrelor ADE identification from the FAERS database between 1 October 2010 and 31 March 2023. ADE, adverse drug event; ROR, reporting odds ratio; PRR, proportional reporting ratio; BCPNN, bayesian confidence propagation neural network.

In this study, ROR, PRR and BCPNN were used to analyze ADE signals, and 263 risk signals were detected. Furthermore, 22 invalid signals were eliminated including non-reference value ADEs (e.g., inability to afford medication, insurance issues), ADEs related to primary diseases (e.g., acute myocardial infarction, unstable angina pectoris, ACS), and drug use error events (e.g., product name confusion, intentional product misuse), shown in Supplementary Table S3. Finally, 241 positive signals by three methods were included (Figure 1).

### 3.3 SOCs of ADE signals

The 241 positive ADE signals were classified using MedDRA for the involved organs and systems. A total of 18 SOCs were involved in ticagrelor ADE signals. The common SOCs were respiratory, thoracic, and mediastinal disorders (2,637 reports, 23 signals), general disorders and administration site conditions (1,688 reports, 13 signals), gastrointestinal disorders (1,131 reports, 39 signals), and cardiac disorders (1,050 reports, 45 signals) (Supplementary Table S4).

### 3.4 ADE frequency analysis

The top 50 ADEs of ticagrelor based on the number of reports are shown in Table 2. The main ticagrelor-related ADEs were dyspnea-related, bleeding-related, and bradycardia-related ADEs. Dyspnea and hemorrhage were the most serious ADEs with high signal strength mentioned in the package insert. Some ADEs not mentioned in the package insert, such as chest pain, feeling abnormal, chest discomfort, cardiac arrest, and thrombosis, were found to be possible new ADE signals.

**TABLE 2:** PT signal detection results of the top 50 ADEs based on the number of reports about ticagrelor.

PTs	Reports	ROR (95% CI)	PRR (χ^2^)	IC (IC025)
Dyspnea	1824	7.34(6.99–7.72)	6.45(8,505.60)	2.68(2.60)
Chest pain^a^	458	5.43(4.94–5.96)	5.27(1,579.28)	2.39(2.24)
Vascular stent thrombosis	406	409.53(362.66–462.45)	396.68(104,579.20)	8.02(7.14)
Contusion	399	8.63(7.81–9.54)	8.39(2,571.97)	3.06(2.88)
Hemorrhage	399	6.56(5.93–7.25)	6.39(1,800.29)	2.66(2.50)
Feeling abnormal^a^	326	2.34(2.09–2.61)	2.30(241.32)	1.20(1.03)
Chest discomfort^a^	283	6.09(5.41–6.86)	5.98(1,164)	2.57(2.37)
Epistaxis	270	6.98(6.18–7.88)	6.86(1,336.38)	2.77(2.56)
Gastrointestinal hemorrhage	270	5.00(4.43–5.64)	4.91(835.45)	2.29(2.09)
Anemia	250	2.81(2.48–3.19)	2.78(283.63)	1.47(1.28)
Cerebral hemorrhage	169	8.43(7.24–9.82)	8.33(1,073.04)	3.04(2.76)
Hemoglobin decreased	152	3.31(2.82–3.89)	3.29(239.22)	1.71(1.46)
Cardiac arrest^a^	137	3.41(2.88–4.04)	3.39(227.59)	1.75(1.48)
Thrombosis^a^	132	3.00(2.53–3.56)	2.98(171.44)	1.57(1.30)
Intracranial hemorrhage	123	12.42(10.38–14.85)	12.31(1,247.09)	3.60(3.21)
Bradycardia	123	4.35(3.64–5.20)	4.32(309.49)	2.10(1.81)
Melaena	108	9.31(7.69–11.26)	9.24(776.16)	3.19(2.81)
Syncope	105	2.06(1.70–2.50)	2.05(55.66)	1.04(0.74)
Asphyxia^a^	101	23.60(19.35–28.80)	23.43(2,081.81)	4.51(3.94)
Atrial fibrillation^a^	99	2.05(1.68–2.50)	2.04(51.51)	1.03(0.72)
Hypoacusis^a^	94	2.85(2.33–3.50)	2.84(110.11)	1.50(1.18)
Rectal hemorrhage	83	4.02(3.24–4.99)	4.00(182.89)	1.99(1.63)
Hematuria	81	4.43(3.56–5.51)	4.41(209.04)	2.13(1.75)
Stress^a^	81	2.17(1.74–2.70)	2.16(49.50)	1.11(0.77)
Vascular stent occlusion	71	630.24(459.67–864.11)	626.78(23,830.40)	8.42(5.48)
Vascular stent stenosis	68	185.18(141.95–241.56)	184.21(9,801.84)	7.21(5.18)
Faces discolored	68	6.64(5.22–8.43)	6.61(315.45)	2.71(2.25)
Blood pressure decreased^a^	64	2.09(1.63–2.67)	2.08(34.84)	1.05(0.67)
Exertional dyspnea	63	3.94(3.07–5.05)	3.92(133.75)	1.97(1.54)
Hematochezia	61	2.40(1.86–3.08)	2.39(47.79)	1.25(0.85)
Hematemesis	58	4.73(3.65–6.13)	4.71(165.07)	2.23(1.76)
Nervousness^a^	58	2.06(1.59–2.67)	2.05(30.31)	1.04(0.63)
Rhabdomyolysis^a^	57	2.75(2.12–3.57)	2.75(61.38)	1.45(1.03)
Cardio-respiratory arrest^a^	55	2.61(2.00–3.4)	2.60(52.50)	1.38(0.95)
Hematoma	52	3.93(2.99–5.16)	3.92(109.55)	1.96(1.49)
Hemoptysis	52	3.88(2.96–5.1)	3.87(107.43)	1.95(1.47)
Pulmonary edema^a^	52	2.45(1.87–3.22)	2.45(43.03)	1.29(0.85)
Upper gastrointestinal hemorrhage	51	5.49(4.16–7.23)	5.47(180.63)	2.44(1.92)
Complete atrioventricular block^a^	48	14.73(11.06–19.61)	14.68(586.83)	3.85(3.08)
Gastric ulcer^a^	47	5.01(3.76–6.68)	5.00(145.33)	2.31(1.78)
Hemorrhagic stroke	46	10.76(8.04–14.41)	10.73(390.71)	3.40(2.71)
Subdural hematoma	46	5.64(4.22–7.54)	5.62(168.99)	2.48(1.92)
Gout	46	5.15(3.85–6.88)	5.13(147.98)	2.35(1.81)
Visual acuity reduced^a^	45	2.51(1.88–3.37)	2.51(39.23)	1.32(0.85)
Atrioventricular block^a^	44	10.70(7.94–14.42)	10.67(370.73)	3.40(2.69)
Skin discoloration^a^	44	2.02(1.50–2.71)	2.01(21.36)	1.01(0.54)
Gastric hemorrhage	43	5.74(4.25–7.76)	5.73(161.97)	2.51(1.93)
Cardiogenic shock^a^	42	5.86(4.32–7.94)	5.84(162.51)	2.54(1.94)
Sleep apnea syndrome	42	4.49(3.31–6.08)	4.48(109.30)	2.16(1.60)
Sinus arrest^a^	41	58.67(42.68–80.66)	58.49(2,096.27)	5.76(4.12)

^a^ ADE, not recorded in the drug labels/datasheets; ADE, adverse drug event; PTs, preferred terms; ROR, reporting odds ratio; CI, confidence interval; PRR, proportional reporting ratio; χ2, chi-squared; IC, information component; IC025, the lower limit of 95%CI, of the IC.

The top 50 ADEs of ticagrelor based on risk strength are shown in Table 3. The ADEs with high signal intensity are mainly bradycardia-related ADEs. Among them, ventricular asystole [16 reports, ROR 218.56, 95% confidence interval (CI) 125.22–381.48], Cheyne-Stokes respiration (8 reports, ROR 87.05, 95% CI 41.86–181.05), and sinoatrial block (19 reports, ROR 63.74, 95% CI 39.89–101.85) had strong ADE signals. In addition, ADEs such as ventricular asystole, sinoatrial block, sinus arrest, idioventricular rhythm, and a feeling of suffocation were not mentioned in the package insert.

**TABLE 3:** PT signal detection results of top 50 ADEs based on signal strength about ticagrelor.

PTs	Reports	ROR (95% CI)	PRR (χ^2^)	IC (IC025)
Vascular stent occlusion	71	630.24(459.67–864.11)	626.78(23,830.40)	8.42(5.48)
Vascular stent thrombosis	406	409.53(362.66–462.45)	396.68(104,579.20)	8.02(7.14)
Arterial restenosis	18	375.71(213.31–661.74)	375.19(4,231.97)	7.97(3.38)
Restenosis	4	250.20(80.68–775.88)	250.12(569.05)	7.55(0.82)
Ventricular asystole^a^	16	218.56(125.22–381.48)	218.29(2,514.95)	7.40(3.18)
Vascular stent stenosis	68	185.18(141.95–241.56)	184.21(9,801.84)	7.21(5.18)
Coronary artery restenosis	27	143.99(95.36–217.42)	143.69(3,092.17)	6.92(3.93)
Coronary artery reocclusion	5	125.11(48.53–322.51)	125.06(426.34)	6.75(1.24)
Platelet function test abnormal	12	106.03(57.92–194.11)	105.94(1,002.89)	6.54(2.67)
Cheyne-stokes respiration	8	87.05(41.86–181.05)	87.00(534.71)	6.29(2.01)
Sinoatrial block^a^	19	63.74(39.89–101.85)	63.65(1,022.97)	5.88(3.24)
Sinus arrest^a^	41	58.67(42.68–80.66)	58.49(2,096.27)	5.76(4.12)
Idioventricular rhythm^a^	9	52.80(26.85–103.81)	52.76(379.95)	5.63(2.13)
Vascular occlusion	35	36.02(25.65–50.59)	35.93(1,101.09)	5.10(3.66)
Vascular stenosis	5	32.36(13.22–79.22)	32.34(117.06)	4.96(1.16)
A feeling of suffocation^a^	34	31.46(22.31–44.36)	31.38(930.99)	4.91(3.54)
Spinal cord hematoma	5	30.27(12.38–74.02)	30.26(109.27)	4.86(1.15)
Bleeding time prolonged	17	28.51(17.56–46.29)	28.47(408.10)	4.78(2.78)
Gastrointestinal angiodysplasia^a^	6	28.33(12.54–64.02)	28.32(127.14)	4.77(1.41)
Cardiac ventricular thrombosis	13	25.36(14.59–44.09)	25.34(270.88)	4.62(2.41)
Asphyxia^a^	101	23.60(19.35–28.80)	23.43(2,081.81)	4.51(3.94)
Coronary artery stenosis	38	22.73(16.45–31.40)	22.67(743.10)	4.46(3.37)
Occult blood	5	19.25(7.92–46.77)	19.24(67.43)	4.23(1.05)
Microcytic anemia	16	17.97(10.94–29.51)	17.95(233.77)	4.13(2.44)
Vascular pseudoaneurysm^a^	15	16.14(9.68–26.93)	16.13(193.79)	3.98(2.31)
Cardiac aneurysm^a^	7	15.55(7.35–32.87)	15.54(79.58)	3.93(1.42)
Traumatic intracranial hemorrhage	8	15.36(7.63–30.94)	15.35(91.58)	3.91(1.58)
Erosive duodenitis^a^	6	15.32(6.83–34.39)	15.31(65.25)	3.91(1.22)
Complete atrioventricular block^a^	48	14.73(11.06–19.61)	14.68(586.83)	3.85(3.08)
Coronary artery dissection^a^	7	13.94(6.60–29.45)	13.93(70.28)	3.78(1.37)
Orthopnea^a^	19	13.52(8.59–21.29)	13.50(203.94)	3.73(2.39)
Paroxysmal nocturnal dyspnea	4	12.78(4.76–34.33)	12.77(31.87)	3.65(0.62)
Intracranial hemorrhage	123	12.42(10.38–14.85)	12.31(1,247.09)	3.60(3.21)
Nocturnal dyspnea	7	12.31(5.83–25.98)	12.30(60.81)	3.60(1.31)
Myocardial necrosis marker increased	12	12.28(6.94–21.73)	12.27(111.35)	3.60(1.90)
Subcutaneous hematoma	8	12.18(6.06–24.50)	12.18(70.12)	3.58(1.46)
Gastrointestinal polyp hemorrhage	4	12.11(4.51–32.52)	12.10(29.90)	3.58(0.60)
Retroperitoneal hematoma	14	11.92(7.03–20.22)	11.91(127.21)	3.55(2.02)
Atrioventricular block second degree^a^	18	11.73(7.36–18.69)	11.71(163.30)	3.53(2.22)
Irregular breathing^a^	4	11.46(4.27–30.77)	11.46(28.00)	3.50(0.58)
Ear hemorrhage	11	11.25(6.20–20.42)	11.25(91.31)	3.47(1.75)
Cerebral mass effect^a^	6	11.20(5.00–25.09)	11.20(45.31)	3.47(1.08)
Cerebellar hemorrhage	9	11.02(5.71–21.29)	11.02(71.20)	3.44(1.53)
Acute left ventricular failure^a^	4	10.92(4.07–29.30)	10.91(26.40)	3.43(0.56)
Hemorrhagic stroke	46	10.76(8.04–14.41)	10.73(390.71)	3.40(2.71)
Bradyarrhythmia	9	10.71(5.55–20.68)	10.70(68.77)	3.40(1.51)
Atrioventricular block^a^	44	10.70(7.94–14.42)	10.67(370.73)	3.40(2.69)
Brain death^a^	16	10.67(6.52–17.49)	10.66(128.78)	3.40(2.05)
Dyspnea at rest	13	10.38(6.00–17.94)	10.37(99.47)	3.36(1.85)
Iron deficiency anemia	37	10.01(7.23–13.85)	9.98(286.31)	3.30(2.53)

^a^ ADE, not recorded in the drug labels/datasheets; ADE, adverse drug event; PTs, preferred terms; ROR, reporting odds ratio; CI, confidence interval; PRR, proportional reporting ratio; χ2, chi-squared; IC, information component; IC025, the lower limit of 95%CI, of the IC.

### 3.5 Bleeding-related PT

In this study, we found bleeding-related ADEs of ticagrelor distributed to 14 SOCs (Supplementary Table S5). The highest numbers of reports and signals were found for gastrointestinal disorders (849 reports, 22 signals), followed by injury, poisoning, and procedural complications (548 reports, 10 signals) and vascular disorders (520 reports, 4 signals). The PT distribution of the top 20 ADEs based on the number of reports of ticagrelor-related hemorrhage is shown in Table 4.

**TABLE 4:** PT signal detection results of top 20 ADEs related to hemorrhage induced by ticagrelor.

PTs	Reports	ROR (95% CI)	PRR (χ^2^)	IC (IC025)	Intensity
Contusion	399	8.63(7.81–9.54)	8.39(2,571.97)	3.06(2.88)	+
Hemorrhage	399	6.56(5.93–7.25)	6.39(1,800.29)	2.66(2.50)	+
Gastrointestinal hemorrhage	270	5.00(4.43–5.64)	4.91(835.45)	2.29(2.09)	+
Epistaxis	270	6.98(6.18–7.88)	6.86(1,336.38)	2.77(2.56)	+
Anemia	250	2.81(2.48–3.19)	2.78(283.63)	1.47(1.28)	+
Cerebral hemorrhage	169	8.43(7.24–9.82)	8.33(1,073.04)	3.04(2.76)	+
Hemoglobin decreased	152	3.31(2.82–3.89)	3.29(239.22)	1.71(1.46)	+
Intracranial hemorrhage	123	12.42(10.38–14.85)	12.31(1,247.09)	3.60(3.21)	+ +
Melaena	108	9.31(7.69–11.26)	9.24(776.16)	3.19(2.81)	+
Rectal hemorrhage	83	4.02(3.24–4.99)	4.00(182.89)	1.99(1.63)	+
Hematuria	81	4.43(3.56–5.51)	4.41(209.04)	2.13(1.75)	+
Faces discolored	68	6.64(5.22–8.43)	6.61(315.45)	2.71(2.25)	+
Hematochezia	61	2.40(1.86–3.08)	2.39(47.79)	1.25(0.85)	+
Hematoma	52	3.93(2.99–5.16)	3.92(109.55)	1.96(1.49)	+
Hemoptysis	52	3.88(2.96–5.10)	3.87(107.43)	1.95(1.47)	+
Upper gastrointestinal hemorrhage	51	5.49(4.16–7.23)	5.47(180.63)	2.44(1.92)	+
Subdural hematoma	46	5.64(4.22–7.54)	5.62(168.99)	2.48(1.92)	+
Hemorrhagic stroke	46	10.76(8.04–14.41)	10.73(390.71)	3.40(2.71)	+ +
Gastric hemorrhage	43	5.74(4.25–7.76)	5.73(161.97)	2.51(1.93)	+
Internal hemorrhage	39	4.32(3.16–5.93)	4.31(95.51)	2.10(1.53)	+

PTs, preferred terms; ROR, reporting odds ratio; CI, confidence interval; PRR, proportional reporting ratio; χ2, chi-squared; IC, information component; IC025, the lower limit of 95%CI, of the IC; intensity, the judgment criteria of signal intensity was shown in Supplementary Table S1.

### 3.6 PTs in respiratory, thoracic and mediastinal disorders

PTs related to respiratory, thoracic, and mediastinal disorders are shown in Table 5. We identified new ADEs added to FDA-approved drug instructions, such as sleep apnea syndrome (42 reports, ROR 4.49, 95% CI 3.31–6.08) and Cheyne-Stokes respiration (8 reports, ROR 87.05, 95% CI 41.86–181.05) which have extremely high signal strength. Asphyxia, a feeling of suffocation, tachypnea, bronchospasm, orthopnea, hyperventilation, abnormal respiration, apnea, and acute pulmonary edema were found as possible new ADE risk signals.

**TABLE 5:** PT signal detection results of top 20 ADEs related to respiratory, thoracic, and mediastinal disorders induced by ticagrelor.

PTs	Reports	ROR (95% CI)	PRR (χ^2^)	IC (IC025)	Intensity
Dyspnea	1824	7.34(6.99–7.72)	6.45(8,505.6)	2.68(2.60)	+
Epistaxis	270	6.98(6.18–7.88)	6.86(1,336.38)	2.77(2.56)	+
Asphyxia^a^	101	23.60(19.35–28.80)	23.43(2081.81)	4.51(3.94)	+ +
Exertional dyspnea	63	3.94(3.07–5.05)	3.92(133.75)	1.97(1.54)	+
Hemoptysis	52	3.88(2.96–5.10)	3.87(107.43)	1.95(1.47)	+ +
Pulmonary edema	52	2.45(1.87–3.22)	2.45(43.03)	1.29(0.85)	+
Sleep apnea syndrome	42	4.49(3.31–6.08)	4.48(109.30)	2.16(1.60)	+
A feeling of suffocation^a^	34	31.46(22.31–44.36)	31.38(930.99)	4.91(3.54)	+ +
Pulmonary hemorrhage	32	8.40(5.93–11.91)	8.38(198.53)	3.05(2.26)	+
Tachypnea^a^	29	4.52(3.13–6.51)	4.51(75.31)	2.17(1.48)	+
Bronchospasm^a^	20	3.39(2.19–5.27)	3.39(31.22)	1.76(0.97)	+
Orthopnea^a^	19	13.52(8.59–21.29)	13.5(203.94)	3.73(2.39)	+ +
Hyperventilation^a^	14	5.17(3.06–8.75)	5.17(42.69)	2.36(1.26)	+
Dyspnea at rest	13	10.38(6.00–17.94)	10.37(99.47)	3.36(1.85)	+ +
Pulmonary alveolar hemorrhage	12	4.44(2.52–7.83)	4.44(28.42)	2.14(1.00)	+
Respiration abnormal^a^	12	3.32(1.88–5.85)	3.31(17.06)	1.72(0.69)	+
Apnea^a^	12	3.15(1.79–5.55)	3.15(15.43)	1.65(0.63)	+
Cheyne-stokes respiration	8	87.05(41.86–181.05)	87.00(534.71)	6.29(2.01)	+ + +
Acute pulmonary edema^a^	8	3.74(1.87–7.49)	3.74(13.34)	1.90(0.55)	+
Nocturnal dyspnea	7	12.31(5.83–25.98)	12.30(60.81)	3.60(1.31)	+ +

^a^ ADE, not recorded in the drug labels/datasheets; PTs, preferred terms; ROR, reporting odds ratio; CI, confidence interval; PRR, proportional reporting ratio; χ2, chi-squared; IC, information component; IC025, the lower limit of 95%CI, of the IC; intensity, the judgment criteria of signal intensity was shown in Supplementary Table S1.

### 3.7 PTs in cardiac disorders

PTs related to cardiac disorders are shown in Table 6. We identified new ADEs added to the FDA-approved drug instructions, such as bradycardia (123 reports, ROR 4.35, 95% CI 3.64–5.20), which had an extremely high signal strength. Cardiac arrest, atrial fibrillation, cardio-respiratory arrest, atrioventricular block complete, atrioventricular block, and cardiogenic shock were found as possible new ADE risk signals.

**TABLE 6:** PT signal detection results of top 20 ADEs related to cardiac disorders induced by ticagrelor.

PTs	Reports	ROR (95% CI)	PRR (χ^2^)	IC (IC025)	Intensity
Cardiac arrest^a^	137	3.41(2.88–4.04)	3.39(227.59)	1.75(1.48)	+
Bradycardia	123	4.35(3.64–5.20)	4.32(309.49)	2.10(1.81)	+
Atrial fibrillation^a^	99	2.05(1.68–2.50)	2.04(51.51)	1.03(0.72)	+
Cardio-respiratory arrest^a^	55	2.61(2.00–3.40)	2.60(52.50)	1.38(0.95)	+
Complete atrioventricular block^a^	48	14.73(11.06–19.61)	14.68(586.83)	3.85(3.08)	+ +
Atrioventricular block^a^	44	10.70(7.94–14.42)	10.67(370.73)	3.40(2.69)	+ +
Cardiogenic shock^a^	42	5.86(4.32–7.94)	5.84(162.51)	2.54(1.94)	+
Sinus arrest^a^	41	58.67(42.68–80.66)	58.49(2,096.27)	5.76(4.12)	+ +
Coronary artery stenosis	38	22.73(16.45–31.40)	22.67(743.1)	4.46(3.37)	+ + +
Pericardial effusion^a^	32	2.87(2.03–4.06)	2.86(36.80)	1.51(0.93)	+
Ventricular fibrillation^a^	28	5.52(3.81–8.01)	5.51(98.29)	2.45(1.71)	+
Myocardial ischemia^a^	26	4.50(3.06–6.62)	4.49(66.79)	2.16(1.43)	+
Ventricular tachycardia^a^	26	3.37(2.29–4.96)	3.37(40.80)	1.75(1.07)	+
Sinoatrial block^a^	19	63.74(39.89–101.85)	63.65(1,022.97)	5.88(3.24)	+ + +
Atrioventricular block second degree^a^	18	11.73(7.36–18.69)	11.71(163.30)	3.53(2.22)	+ +
Sinus bradycardia^a^	18	3.73(2.34–5.92)	3.72(32.99)	1.89(1.03)	+
Ventricular asystole^a^	16	218.56(125.22–381.48)	218.29(2,514.95)	7.40(3.18)	+ + +
Cardiac ventricular thrombosis	13	25.36(14.59–44.09)	25.34(270.88)	4.62(2.41)	+ +
Cardiac tamponade^a^	13	4.90(2.84–8.46)	4.90(36.31)	2.29(1.16)	+
Cardiac failure acute^a^	13	4.15(2.41–7.16)	4.15(27.81)	2.05(0.98)	+ +

^a^ ADE, not recorded in the drug labels/datasheets; PTs, preferred terms; ROR, reporting odds ratio; CI, confidence interval; PRR, proportional reporting ratio; χ2.

chi-squared; IC, information component; IC025, the lower limit of 95%CI, of the IC; intensity, the judgment criteria of signal intensity was shown in Supplementary Table S1.

## 4 Discussion

In this study, bleeding, dyspnea, and bradycardia-related ADEs were the main ADEs of ticagrelor. Due to differences in the definition of bleeding, the incidence of bleeding induced by ticagrelor reported in previous literature varies widely, fluctuating between 3% and 32% (Wang et al., 2018). The risk of bleeding of ACS patients treated with ticagrelor versus clopidogrel was analyzed in the PLATO study, which showed there was no significant increase in the overall rate of major bleeding (11.6% with ticagrelor and 11.2% with clopidogrel, respectively; *p* = 0.43) (Wallentin et al., 2009). The PEGASUS-TIMI 54 study reported an increased risk of major bleeding with ticagrelor compared to aspirin-backed placebo in patients with prior myocardial infarction over 1 year, but the incidence of TIMI major bleeding was similar between different dosing groups (2.60% in the 90 mg group and 2.30% in the 60 mg group), and the incidence of intracranial or fatal bleeding was 0.63% and 0.71%, which was close to 0.60% in the placebo group (Bonaca et al., 2015). The risk of bleeding is included in the black box warning on the package insert of ticagrelor, and intracranial hemorrhage is defined as the main fatal/life-threatening bleeding in the PLATO trial. This study found that risk signals in the nervous system, including cerebral hemorrhage (169 reports, ROR 8.43, 95% CI 7.24–9.82), intracranial hemorrhage (123 reports, ROR 12.42, 95% CI 10.38–14.85), and hemorrhagic stroke (46 reports, ROR 10.76, 95% CI 8.04–14.41), had a high signal strength. Ticagrelor increases the risk of bleeding while reducing the risk of ischemia, so it is necessary to optimize the balance between ischemia and bleeding risk. Therefore, the dosage and duration of ticagrelor should be evaluated individually based on the patient’s risk of ischemia and bleeding, the occurrence of adverse events, complications, and combination with other drugs.

Gastrointestinal disorders had the third highest number of PT signals (1,131 reports, 39 signals), while in the subgroup analysis of bleeding-related ADEs, the highest number of reports and signals was also found for gastrointestinal disorders (849 reports, 22 signals). This is consistent with the observation in clinical studies that the increased risk of bleeding is primarily due to gastrointestinal bleeding (GIB) events, which occur more frequently than other major bleeding events. A meta-analysis showed an increased risk of GIB with third-generation P2Y12 inhibitors compared to clopidogrel (RR = 1.28, 95% CI = 1.13–1.46) and a higher risk of GIB occurring in the upper gastrointestinal tract compared with other sites; with a GIB incidence of 1.25% (216/17329) for ticagrelor, there was no increased risk of GIB compared with clopidogrel (RR = 1.15, 95% CI = 0.94–1.39) (Guo et al., 2019). The results of this study again validate that there is a higher risk of bleeding with a greater proportion originating from the gastrointestinal tract with ticagrelor, which is generally consistent with the results of other studies and the dosing cautionary information. In addition, a clinical safety review by the FDA reported a higher incidence of gastrointestinal AEs with ticagrelor compared to clopidogrel, including overall gastrointestinal or anal bleeding events, spontaneous GIB events, and nausea, vomiting, dyspepsia, diarrhea, and the presence of *Helicobacter pylori*, and a higher incidence of constipation with clopidogrel (Serebruany et al., 2013). This study found risk signals such as gastrointestinal hemorrhage (270 reports, ROR 5.00, 95% CI 4.43–5.64), melaena (108 reports, ROR 9.31, 95% CI 7.69–11.26), and rectal hemorrhage (83 reports, ROR 4.02, 95% CI 3.24–4.99), but the number of reports of nausea, vomiting, dyspepsia, and diarrhea was low, and no signal was detected. This may be because these digestive ADE symptoms are mild and non-specific in patients on multiple medications, and they may be underreported, leading to confounding bias. For patients with a history of GIB and an increased risk of bleeding, ticagrelor should be prescribed with caution, and antiplatelet therapy with clopidogrel or the addition of a proton pump inhibitor for GIB prophylaxis is recommended.

In addition, dyspnea (1824 reports, ROR 7.34, 95% CI 6.99–7.72) was the most reported with strong signal values. The incidence of dyspnea in clinical trials is reported in the package insert as approximately 14%–21%. Adverse reactions of dyspnea (including dyspnea, dyspnea at rest, exertional dyspnea, paroxysmal nocturnal dyspnea, and nocturnal dyspnea) were reported in 13.8% and 7.8% of patients in the ticagrelor and clopidogrel groups, respectively, in the PLATO study (Wallentin et al., 2009). This study revealed dyspnea-related PTs that were generally consistent with the ADEs reported in the PLATO study, in addition to ADEs not included in the package insert, such as asphyxia, a feeling of suffocation, and tachypnea. The PEGASUS-TIMI 54 study reported more frequent dyspnea in both ticagrelor dose groups compared with the aspirin-backed placebo group, with a slightly lower incidence in the low-dose group than in the high-dose group (18.93% in the 90 mg group and 15.84% in the 60 mg group). Most episodes of dyspnea were mild (58.1%) or moderate (36.9%) in severity, mostly single episodes early after treatment initiation, which resolved spontaneously or after discontinuation of the drug (Bonaca et al., 2015). The PLATO study showed that, compared with patients in the clopidogrel group, patients with dyspnea in the ticagrelor group were more likely to have onset of dyspnea within 7 days, with a median duration of 23 days (Storey et al., 2011). The mechanism of ticagrelor-related dyspnea remains to be confirmed, and current studies suggest that dyspnea is most often seen with reversible P2Y12 inhibitors. Moreover, by analyzing the ADEs of ticagrelor associated with the respiratory system, we identified risk signals for sleep apnea syndrome (42 reports, ROR 4.49, 95% CI 3.31–6.08), apnea (12 reports, ROR 3.15, 95% CI 1.79–5.55), and Cheyne-Stokes respiration (8 reports, ROR 87.05, 95% CI 41.86–181.05). A previous study using the VigiBase database found 28 cases of sleep apnea in ADE reports associated with ticagrelor, and through a proportional imbalance analysis, sleep apnea was identified as a risk signal for ticagrelor (ROR = 4.16, 95% CI = 2.87–6.03) (Revol et al., 2018). A single-center prospective clinical trial was conducted to assess the association between CSA hypoventilation syndrome (CSAHS) and ticagrelor administration; a high prevalence of CSA after ACS was found (22.3%), and a much higher incidence was found in patients treated with ticagrelor than in those who were not (30% vs. 7.3%), confirming the association between ticagrelor and CSA (Meurin et al., 2021). As a result, the US FDA approved a new safety statement about CSA and Cheyne-Stokes respiration in September 2020 (FDA, 2020). Current hypotheses of underlying mechanisms of dyspnea related adverse reactions caused by ticagrelor include the antagonism of microglial P2Y12 receptors (Revol et al., 2018), the inhibition of the type 1 equilibrative nucleoside transporter (ENT1) protein and its effects on tissue adenosine levels or the inhibition of P2Y12 receptors located on C fibres of sensory neurons (Parodi and Storey, 2015). Therefore, caution is advised in patients with a history of asthma/chronic obstructive pulmonary disease; if dyspnea occurs during dosing, first assess its severity, whether it worsens, and whether it is due to the original disease or other causes; if the symptoms are mild and tolerated by the patient, continue to use ticagrelor and monitor the patient closely; if dyspnea worsens or is not tolerated by the patient and is suspected to be caused by ticagrelor, a switch to clopidogrel can be made.

The present study revealed many PTs that were positively related to arrhythmia; PTs related to bradycardia include cardiac arrest (137 reports, ROR 3.41, 95% CI 2.88–4.04), bradycardia (123 reports, ROR 4.35, 95% CI 3.64–5.20), complete atrioventricular block (48 reports, ROR 14.73, 95% CI 11.06–19.61), atrioventricular block (44 reports, ROR 10.70, 95% CI 7.94–14.42), and sinus arrest (41 reports, ROR 58.67, 95% CI 42.68–80.66). Among them, bradycardia is mentioned in the drug labels/datasheets. There are multiple case reports about bradycardia-related ADEs caused by ticagrelor and serious bradyarrhythmia, both as early effects or in a delayed fashion (Al-Bayati et al., 2021; Aranganathan et al., 2021; Kotaru and Kalavakunta, 2021). The EMA identified ticagrelor-related bradyarrhythmia as a potential safety issue and included it in the European Risk Management Plan in 2011 (Pujade et al., 2020). In the DISPERSE-2 clinical trial (Cannon et al., 2007), ventricular pauses were observed in patients receiving ticagrelor treatment. Therefore, the PLATO, PEGASUS, THEMIS, and THALES trials excluded patients at increased risk of bradycardic events (e.g., patients who have sick sinus syndrome, second or third-degree AV block, or bradycardia-related syncope and are not protected with a pacemaker) (Scirica et al., 2011; Steg et al., 2019; Johnston et al., 2020; Bergmark et al., 2021). The electrocardiographic (ECG) sub-study of PLATO showed that ticagrelor compared to clopidogrel did not increase arrhythmic events even in subjects with ACS who present with mild conduction abnormalities on their baseline ECG (Scirica et al., 2018). However, a current meta-analysis of randomized controlled trials found an increased risk of both bradyarrhythmia and severe bradyarrhythmia; the latter seems mostly due to ventricular pauses of >2.5 s, but due to the lack of outcome data in two-thirds of eligible studies, the evidence is low to moderate (Pujade et al., 2020). The mechanism underlying ticagrelor-induced bradycardia is incompletely understood. On the one hand, ticagrelor may increase adenosine levels by inhibiting cellular adenosine uptake through the ENT1 transporter, causing bradycardia and heart block. On the other hand, ticagrelor may have a direct effect on automaticity and cardiac conduction (Cattaneo et al., 2014). In addition, ticagrelor seems to also increase the risk of tachycardia. Atrial fibrillation (99 reports, ROR 2.05, 95% CI 1.68–2.50), ventricular fibrillation (28 reports, ROR 5.52, 95% CI 3.81–8.01), and ventricular tachycardia (26 reports, ROR 3.73, 95% CI 2.29–4.96) were positive signals related with tachycardia. At present, there is no consensus on whether ticagrelor can cause tachycardia. A case report described a patient with unstable angina pectoris and a history of paroxysmal atrial fibrillation developed recurrent atrial fibrillation following the use of ticagrelor (Zhang et al., 2016). Ticagrelor could increase the adenosine half-life and plasma concentration levels and enhance the biological effects of adenosine, which has the potential to cause atrial fibrillation(Akkaif et al., 2021). However, a cross-sectional study did not find any difference in detailed ECG and echocardiographic parameters as atrial fibrillation predictors between ticagrelor and clopidogrel groups in ACS patients (Algül et al., 2019). Therefore, it is necessary to be aware that bradycardia may be related to the use of ticagrelor. Patients with bradycardia risk factors should be cautious when using ticagrelor. In addition, after the start of ticagrelor treatment, ACS patients should undergo careful ECG monitoring.

In addition, rhabdomyolysis (57 reports, ROR 2.75, 95% CI 2.12–3.57), which is not mentioned in the package insert, was reported in a high number. After reviewing individual cases, it was found that 52 of the reports received combination therapy with statins. Current national and international guidelines recommend dual antiplatelet therapy and long-term administration of statins for secondary prevention of cardiovascular events for the management of patients with ACS (Amsterdam et al., 2014; Collet et al., 2021). This ADE is most likely the result of a drug-drug interaction (DDI) between ticagrelor and statins because ticagrelor is a CYP3A4 substrate and a weak inhibitor of CYP3A4, which may lead to increased concentrations of statins such as simvastatin, leading to rhabdomyolysis. A pharmacokinetic study in healthy volunteers showed a significant increase in exposure when combined with simvastatin (80 mg) and atorvastatin (80 mg), which are metabolized by CYP3A4, and it is recommended that during treatment with ticagrelor, simvastatin should not be administered at doses greater than 40 mg (Teng et al., 2013). Although the increase in exposure to atorvastatin was modest, the first case of a ticagrelor-atorvastatin interaction was reported. A 62-year-old female patient was diagnosed with rhabdomyolysis after 2 months of treatment with ticagrelor 90 mg twice daily, atorvastatin 80 mg once daily, metoprolol 25 mg twice daily, and aspirin. Kido et al. considered it might be related to the use of ticagrelor (Kido et al., 2015). Although Rosuvastatin is mainly metabolized by CYP2C9, there are reports of rhabdomyolysis caused by the DDI of rosuvastatin and ticagrelor. Vrkić Kirhmajer et al. reported 8 cases of rhabdomyolysis caused by the combination of rosuvastatin and ticagrelor as of early 2018 in the WHO Adverse Drug Reaction Database (VigiBase). Three potential mechanisms of action for the occurrence of DDI with ticagrelor and rosuvastatin are also summarized (Vrkić Kirhmajer et al., 2018): (i) renal impairment caused by ticagrelor, leading to reduced renal excretion of rosuvastatin, (ii) competition in the levels of transporter proteins (OATP1B1, P-glycoprotein, ABCG2, MRP2), leading to reduced biliary and renal excretion of rosuvastatin, and (iii) genetic polymorphisms in metabolic enzymes (CYPs, UGTs) and drug transporter proteins, leading to increased competition between drugs. It has been shown that risk factors for rhabdomyolysis include renal impairment, hypertension, diabetes, and older age (Nguyen et al., 2018). For patients with the above risk factors, a combination of lower dose of statins and other lipid-lowering agents may be considered when using ticagrelor or replaced with other antiplatelet agents (Roule et al., 2023). During the initial phase of ticagrelor treatment, close monitoring is required. If rhabdomyolysis occurs, the drug should be stopped immediately, and the antiplatelet drug regimen should be changed.

The following limitations exist in this study. First, we found the top number of reports for “vascular disorders” and “general disorders and administration site conditions” according to SOC classification, including many ADEs that may be related to the progression of primary diseases such as vascular stent occlusion, vascular stent thrombosis, arterial restenosis, and restenosis. It is important to highlight that patients using ticagrelor face an elevated risk of encountering symptoms such as respiratory distress, stent thrombosis, or chest pain attributed to potential disease effects. These symptoms may be documented as ADEs and result in positive signals. Our study, however, only establishes statistical associations, as the FAERS database lacks a causal relationship between a drug and an ADE. When symptoms associated with the primary disease manifest, it is crucial to provide a meticulous explanation. In addition, FAERS is a self-reporting system, the quality of the reports were unable to be guaranteed and the overall population size using ticagrelor is unknown, underreporting may occur in the ADE reporting process, making it difficult to calculate the incidence of ADEs.

## 5 Conclusion

Our study, analyzing real-world data from the FAERS database, identified 18 System Organ Classes (SOCs) affected by ticagrelor ADEs, predominantly in respiratory, thoracic, and mediastinal systems. Common ADEs like bleeding, dyspnea, and bradycardia were consistent with package insert reports, with notable findings in gastrointestinal bleeding and rare ADEs such as sleep apnea syndrome and Cheyne-Stokes respiration. Additionally, we identified new ADEs including cardiac arrest, atrial fibrillation, asphyxia, and rhabdomyolysis.

## Data Availability

The original contributions presented in the study are included in the article/Supplementary Material, further inquiries can be directed to the corresponding author.
