# Supplementary material for: A disproportionality analysis of FDA adverse event reporting system (FAERS) events for ticagrelor
Source: Front Pharmacol. 2024 Apr 9;15:1251961. doi: 10.3389/fphar.2024.1251961 (PMC11035729; doi:10.3389/fphar.2024.1251961)
Supplement: Supplementary file 1 [file Table1.docx]

Supplementary Material

A Disproportionality Analysis of FDA Adverse Event Reporting System (FAERS) Events for Ticagrelor

Yunyan Pan^1,2†^, Yu Wang^1,2†^, Yifan Zheng^1,3^, Jie Chen ^1^, Jia Li^1*^

^1^Department of Pharmacy, the First Affiliated Hospital of Sun Yat-sen University, Guangzhou 510080, China

^2^School of Pharmaceutical Sciences, Sun Yat-sen University, Guangzhou 510006, China

^3^Department of Clinical Pharmacy Translational Science, University of Michigan College of Pharmacy, Ann Arbor, 48108, USA

*** Correspondence:**Jia Li
[lijia37@mail.sysu.edu.cn](mailto:lijia37@mail.sysu.edu.cn)

^†^These authors contributed equally to this work and share the first authorship.

# Supplementary Figures and Tables

## Supplementary Tables

**Supplementary Table S1. The judgment criterias of risk signal strength for ROR and PRR.**

| Algorithms | Judgment criteria | Signal intensity | Display method |
| --- | --- | --- | --- |
| ROR | 2 ＜ ROR ≤10 | Weak | + |
|  | 10 ＜ ROR ≤50 | Medium | + + |
|  | ROR ＞50 | Strong | + + + |
| PRR | 2 ＜ PRR ≤10 | Weak | + |
|  | 10 ＜ PRR ≤50 | Medium | + + |
|  | PRR ＞ 50 | Strong | + + + |

ROR, reporting odds ratio; PRR, proportional reporting ratio.

**Supplementary Table S2. Basic information on ADE reports related to ticagrelor.**

| Category | | Number of cases | Percentage (%) |
| --- | --- | --- | --- |
| Gender | Male | 7421 | 57.49% |
|  | Women | 4310 | 33.39% |
|  | Unknown | 1178 | 9.13% |
| Age (years) | ＜18 | 13 | 0.10% |
|  | 18~64 | 2661 | 20.61% |
|  | 65~84 | 3302 | 25.58% |
|  | ≥85 | 299 | 2.32% |
|  | Missing | 6634 | 51.39% |
| Year of reporting | 2011 | 64 | 0.50% |
|  | 2012 | 758 | 5.87% |
|  | 2013 | 640 | 4.96% |
|  | 2014 | 781 | 6.05% |
|  | 2015 | 1316 | 10.19% |
|  | 2016 | 3203 | 24.81% |
|  | 2017 | 1407 | 10.90% |
|  | 2018 | 1196 | 9.26% |
|  | 2019 | 986 | 7.64% |
|  | 2020 | 762 | 5.90% |
|  | 2021 | 868 | 6.72% |
|  | 2022 | 716 | 5.55% |
|  | 2023 | 212 | 1.64% |
| Reported countries (top 5) | United States | 8058 | 62.42% |
|  | Russian Federation | 612 | 4.74% |
|  | China | 513 | 3.97% |
|  | Colombia | 348 | 2.70% |
|  | Germany | 320 | 2.48% |
|  | Other | 3058 | 23.69% |

**Supplementary Table S3. The invalid PT signal detection results of ADEs related to ticagrelor.**

| PTs | reports | ROR (95% CI) | PRR (χ^2^) | IC (IC025) |
| --- | --- | --- | --- | --- |
| myocardial infarction | 723 | 8.07(7.48-8.70) | 7.67(4176.26) | 2.93(2.80) |
| intentional product misuse | 261 | 3.77(3.33-4.26) | 3.71(514.25) | 1.89(1.69) |
| thrombosis in device | 237 | 160.45(139.33-184.76) | 157.52(30339.08) | 7.03(6.20) |
| acute myocardial infarction | 235 | 18.26(16.03-20.81) | 17.95(3659.81) | 4.13(3.84) |
| angina pectoris | 124 | 9.53(7.97-11.38) | 9.45(917.32) | 3.22(2.87) |
| coronary artery occlusion | 91 | 18.51(15.03-22.81) | 18.39(1444.30) | 4.17(3.62) |
| unstable angina pectoris | 84 | 31.06(24.96-38.66) | 30.87(2303.53) | 4.89(4.15) |
| device occlusion | 79 | 12.52(10.01-15.64) | 12.45(807.17) | 3.62(3.10) |
| acute coronary syndrome | 75 | 17.37(13.81-21.85) | 17.27(1108.51) | 4.08(3.47) |
| coronary artery disease | 66 | 4.73(3.71-6.03) | 4.71(188.06) | 2.23(1.80) |
| infarction | 61 | 14.02(10.87-18.07) | 13.96(707.80) | 3.78(3.14) |
| coronary artery thrombosis | 33 | 41.65(29.32-59.16) | 41.55(1199.25) | 5.30(3.70) |
| inability to afford medication | 22 | 4.39(2.89-6.68) | 4.39(53.89) | 2.13(1.33) |
| labelled drug-drug interaction medication error | 17 | 2.36(1.47-3.80) | 2.36(11.96) | 1.24(0.45) |
| troponin increased | 16 | 5.24(3.20-8.57) | 5.23(50.27) | 2.38(1.36) |
| insurance issue | 12 | 2.30(1.31-4.06) | 2.30(7.53) | 1.20(0.26) |
| stent malfunction | 10 | 101.48(52.42-196.46) | 101.40(789.45) | 6.48(2.38) |
| product name confusion | 5 | 6.02(2.50-14.53) | 6.02(16.09) | 2.58(0.52) |
| coronary revascularization | 4 | 63.88(23.01-177.33) | 63.86(173.78) | 5.88(0.87) |

*, ADE not recorded in the drug labels/datasheets; ADE, adverse drug event; PTs, preferred terms; ROR, reporting odds ratio; CI, confidence interval; PRR, proportional reporting ratio; χ2, chi-squared; IC, information component; IC025, the lower limit of 95%CI of the IC.

**Supplementary Table S4. System organ class (SOC) distribution: the number of ADE reports and the number of signals for ticagrelor.**

| SOC | ADE reports (%) | ADE signals (%) |
| --- | --- | --- |
| Respiratory, thoracic and mediastinal disorders | 2637(25.4%) | 23(9.5%) |
| General disorders and administration site conditions | 1688(16.3%) | 13(5.4%) |
| Gastrointestinal disorders | 1131(10.9%) | 39(16.2%) |
| Cardiac disorders | 1050(10.1%) | 45(18.7%) |
| Vascular disorders | 811(7.8%) | 18(7.5%) |
| Injury, poisoning and procedural complications | 658(6.3%) | 20(8.3%) |
| Nervous system disorders | 629(6.1%) | 17(7.1%) |
| Investigations | 521(5.0%) | 21(8.7%) |
| Blood and lymphatic system disorders | 433(4.2%) | 13(5.4%) |
| Psychiatric disorders | 219(2.1%) | 8(3.3%) |
| Renal and urinary disorders | 133(1.3%) | 4(1.7%) |
| Ear and labyrinth disorders | 105(1.0%) | 2(0.8%) |
| Skin and subcutaneous tissue disorders | 101(1.0%) | 6(2.5%) |
| Musculoskeletal and connective tissue disorders | 88(0.8%) | 4(1.7%) |
| Eye disorders | 83(0.8%) | 3(1.2%) |
| Metabolism and nutrition disorders | 66(0.6%) | 3(1.2%) |
| Hepatobiliary disorders | 11(0.1%) | 1(0.4%) |
| Reproductive system and breast disorders | 5(＜0.0%) | 1(0.4%) |
| Total | 10369 | 241 |

ADE, adverse drug event

**Supplementary Table S5.** **System organ class (SOC) distribution: the number of ADE reports and the number of signals in ticagrelor-related bleeding ADE reports.**

| SOC | ADE reports (%) | ADE signals (%) |
| --- | --- | --- |
| Gastrointestinal disorders | 849(24.6%) | 22(30.6%) |
| Injury, poisoning and procedural complications | 548(15.9%) | 10(13.9%) |
| Vascular disorders | 520(15.1%) | 4(5.6%) |
| Nervous system disorders | 403(11.7%) | 8(11.1%) |
| Respiratory, thoracic and mediastinal disorders | 371(10.7%) | 5(6.9%) |
| Blood and lymphatic system disorders | 339(9.8%) | 6(8.3%) |
| Investigations | 202(5.9%) | 4(5.6%) |
| Renal and urinary disorders | 103(3%) | 3(4.2%) |
| Skin and subcutaneous tissue disorders | 48(1.4%) | 4(5.6%) |
| Eye disorders | 38(1.1%) | 2(2.8%) |
| Ear and labyrinth disorders | 11(0.3%) | 1(1.4%) |
| Cardiac disorders | 9(0.3%) | 1(1.4%) |
| Musculoskeletal and connective tissue disorders | 6(0.2%) | 1(1.4%) |
| General disorders and administration site conditions | 5(0.1%) | 1(1.4%) |
| Total | 3452 | 72 |

ADE, adverse drug event
